# *Vochysia tucanorum* Mart. butanol fraction presents antitumoral activity in vivo and prevents the installation of cachexia in solid Ehrlich tumor model

**DOI:** 10.1186/s12906-020-03190-1

**Published:** 2021-01-07

**Authors:** Henrique Jorge Novaes Morgan, Aislan Quintiliano Delgado, Luiz Leonardo Saldanha, Nathalia Aparecida De Paula Camaforte, Anne Lígia Dokkedal, José Roberto Bosqueiro

**Affiliations:** 1 Laboratory of Metabolism Control, Ribeirão Preto Medical School, Department of Physiology, University of São Paulo, Ribeirão Preto, State of São Paulo Brazil; 2 Laboratory of Endocrine Pancreas Physiology, Faculty of Science, Department of Physical Education, São Paulo State University, Av. Eng. Luiz Edmundo Carrijo Coube 14-01, Bauru, São Paulo Postal Code: 17033-360 Brazil; 3 Laboratory of Natural Products Chemistry, Faculty of Science, Department of Biological Sciences, São Paulo State University, Bauru, State of São Paulo Brazil

**Keywords:** Anti-apoptotic, Anti-proliferation, Cancer cachexia, Metabolic syndrome, Triterpenes

## Abstract

**Background:**

Cancer is a multifactorial disease caused by uncontrolled proliferation of cells. About 50–80% of cancer patients develop cachexia, a complex metabolic syndrome associated with an increase of mortality and morbidity. However, there are no effective therapies in medical clinic for cancer cachexia. *Vochysia tucanorum* Mart. is a common three of the Brazilian “Cerrado”. The butanolic fraction of *V. tucanorum* (Fr-BuVt), very rich in triterpenes with various biological activities, might be interesting in being tested in cancer cachexia syndrome. Hence, the present study was undertaken to investigate the antitumoral activity of Fr-BuVt and its potential against cachexia development.

**Methods:**

Ehrlich tumor was used as model of cancer cachexia. Ascitic Ehrlich tumor cells were collected, processed and inoculated subcutaneously in saline solution (1 × 10^7^/100 μl; ≥95% viability) for the obtention of solid Ehrlich carcinoma. After inoculation, solid Ehrlich carcinoma-bearing mice were treated by 14 consecutive days by gavage with Fr-BuVt (200 mg/kg). Body weight and tumor volume were measure during the treatment period. Tumors were removed, weighed and properly processed to measure the content and phosphorylation levels of key-proteins involved to apoptotic and proliferation process by Western Blot. Muscles and adipose tissues were removed for weighed. Serum was collected to cytokines levels and energetic blood markers measurements.

**Results:**

The treatment with the Fr-BuVt (200 mg/kg, 14 days) decreased the solid Ehrlich tumor volume and weight besides increased the expression of the pro-apoptotic proteins caspase-3 and BAX, but also decreased the expression of the proteins involved in proliferation NFκB, mTOR and ERK. In addition, our data shows that the administration of Fr-BuVt was able to prevent the installation of cancer cachexia in Ehrlich carcinoma-bearing mice, since prevented the loss of body weight, as well as the loss of muscle and adipose tissue. Moreover, an improvement in some blood parameters such as decrease in cytokines TNF-α and IL-6 levels is observed.

**Conclusions:**

The study revealed that Fr-BuVt has antitumoral activity and prevent installation of cancer cachexia in Ehrlich model. Therefore, Fr-BuVt may represent an alternative treatment for cancer cachexia.

**Supplementary Information:**

The online version contains supplementary material available at 10.1186/s12906-020-03190-1.

## Background

Cancer is the second major cause of death in the world, being a serious public health problem, especially in developing countries [1]. When associated with cachexia, it increases the morbidity and mortality of the patients, besides reduces tolerance to anticancer therapies [2]. Cachexia is a complex metabolic syndrome, which is associated with chronic and immunosuppressive diseases like AIDS, cirrhosis, renal and cardiac insufficiency. However the greatest representativeness is found in cancer patients, since about 80% of them develop this syndrome [3, 4].

Cancer cachexia leads to a negative energy balance and progressive impairment of body function affecting several organs such as muscle, liver, brain, immune system and heart. The substrates from the hypercatabolism of muscle, lipids and carbohydrate are used by both host and tumor, and are the most responsible for the weight loss, the main characteristic of this syndrome [5].

In relation to treatment of neoplasia, cachexia needs multimodal therapies that include nutritional support, physical and medication monitoring, as a way to control morbidity, inflammation and lean and fat mass atrophy. Although, in most cases, therapies against cachexia are inefficient especially when not started in early stages of the disease. However, the diagnosis of this syndrome is not a simple task in the clinic, since there are no good biomarkers to its identification [2, 4, 6]. In addition, the establishment of this syndrome in cancer patients can be due to changes caused by the presence of the tumor as well as by the antineoplastic treatment, being reversed when the therapy is interrupted [3].

In that aspect, we hypothesized that natural products may represent an alternative for the treatment of cancer cachexia since the variety of secondary metabolites present in these extracts can affect multiple metabolic pathways [7].

*Vochysia tucanorum* Mart. (Vochysiaceae) known as “Pau-de-tucano” or “Pau-doce”, presents mainly pentacyclic triterpenes derived from oleanolic acid as previously described [8]. Also, flavones were found in its leaves such as luteolin [9]. This common three of the Brazilian savannah (often called “Cerrado”) is used by traditional communities in South America for the treatment of gastric pain, inflammation, asthma and pulmonary congestion [8].

The pentacyclic triterpenes present in *V. tucanorum* have been described in a series of studies with antitumor and anti-inflammatory activities [10–13]. In vitro, oleanolic acid and ursolic acid were able to increase the DNA fragmentation and activation of caspases pathway leading to an increase of apoptotic process in human hepatic cancer cell lines [14]. In vivo, these triterpenes induced apoptosis through control of cell cycle proteins such as p53 in prostatic cancer xenografts in mice [15]. In addition, pentacyclic terpenoids have been described with anti-inflammatory effects with great potential to treat and prevent cancer and metabolic syndrome [16, 17]. In cachexia perspectivity, triterpenes have potent effects against muscle loss as well as in stimulating muscle growth through enhances in insulin signaling and downregulation of ubiquitin-proteasome pathway [18, 19]. This kind of bioactivity is interesting for cancer therapies as well as might be effective in the treatment of cachexia.

On the other hand, there are few studies addressing the potential of medicinal plants in the treatment of cancer cachexia. In this study, we evaluated the administration of butanolic fraction of *V. tucanorum* Mart. in Ehrlich tumor model of cachexia in mice through molecular, biochemical and clinical parameters analysis. We also evaluated the antitumoral activity of the butanolic fraction.

## Methods

### Plant material and preparations of extract and fraction

Leaves of *V. tucanorum* Mart. were collected in Bauru, State of São Paulo, Brazil (coordinates: 22°20′42.0“S and 49°01’45.5”W), and was identified by Dr. A. L. Dokkedal. A voucher specimen was deposited in the herbarium of the Department of Biological Sciences, School of Sciences, Bauru Campus, São Paulo State University (UNBA) under the number code 5141.

Fresh leaves were separated and dried at 40 °C for 48 h. The powdered leaves were grounded in a knife mill and extracted with EtOH/H_2_O (7:3 *v/v*) by percolation at room temperature. The filtrate was concentrated to dryness under reduced pressure at < 40 °C with yield of 17%. The hydroalcoholic extract was re-suspended in H_2_O and submitted to liquid-liquid extraction with equal volume of *n*-butanol. The *n*-butanol phase was concentrated to dryness under reduced pressure at < 40 °C and lyophylizated resulting in *V. tucanorum n*-butanol fraction (Fr-BuVt). Using this method, the Fr-BuVt was obtained with yield of 3%.

### Identification of constituents by liquid chromatography coupled to mass spectrometry (LC-MS)

The chromatographic profiling of Fr-BuVt was investigated via high performance liquid chromatography (HPLC) (Agilent 1200 Infinity) with quaternary pump and automatic injector (1200 Hip Als), coupled to mass spectrometry (3200 QTRAP® – Linear Ion Trap), AB SCiex. Chromatographic separations were performed using a Luna C-18 (250 × 4.6 mm, 5 μm; Phenomenex) column, conditioned in a column oven at 35 °C. The solvent system was MeOH (solvent A) and H_2_O (solvent B) with 0.1% of formic acid. The gradient was 5–100% of A in B over 60 min.

The ionization via electrospray (Turbo Ion Spray) was performed in negative mode. Ionization source conditions: Ion Spray: − 4500 V, Curtain Gas: 20 psi; Temperature: 650 °C, Gas 1: 50 psi; Gas 2: 50 psi, Interface heater: ON, DP (Declustering Potential) -25.0 V, EP (Entrance Potential) -10.0 V and CEP (Cell entrance potential) -16.0 V. Ion scan mode (EMS – Enhanced Scan): 100–800 Da EPI (Enhanced product ion) Collision energy: 35.0 V +/− 15.0 V. The Fr-BuVT was dissolved in MeOH:H_2_O (1:1, *v/v*), filtered in Polytetrafluoroethylene (PTFE) membrane (0.45 μm) and analysed at a final concentration of 1 ppm.

### Experimental model

Male Swiss mice, 8–12 weeks old were obtained from São Paulo State University (UNESP) facilities and maintained at 22 ± 2 °C under a 12 h light/dark cycle, with free access to food and water.

Ehrlich tumor is a mice spontaneous mammary tumor. It may evolve to ascitic or solid forms depending on the route of inoculation [20]. In this study, the tumor cells were maintained in ascitic form in Swiss mice by intraperitoneal inoculation and collected 7 days after. Ascitic tumor cells were collected by aspiration using a glass pipette, centrifuged for 10 min at 2000 rpm, and washed twice with phosphate-buffered saline (pH 7.4). Cell viability was evaluated by trypan blue exclusion test and only cell suspensions that presented more than 95% viability were used. For solid tumors development, cells (1 × 10^7^/100 μl) were inoculated subcutaneously into the solid Ehrlich tumor groups. After 7 days solid tumor can be observed [21]. For control groups, saline solution (NaCl 0.9%) was subcutaneous injected.

### Treatment with Fr-BuVt

**Fig. 1:**
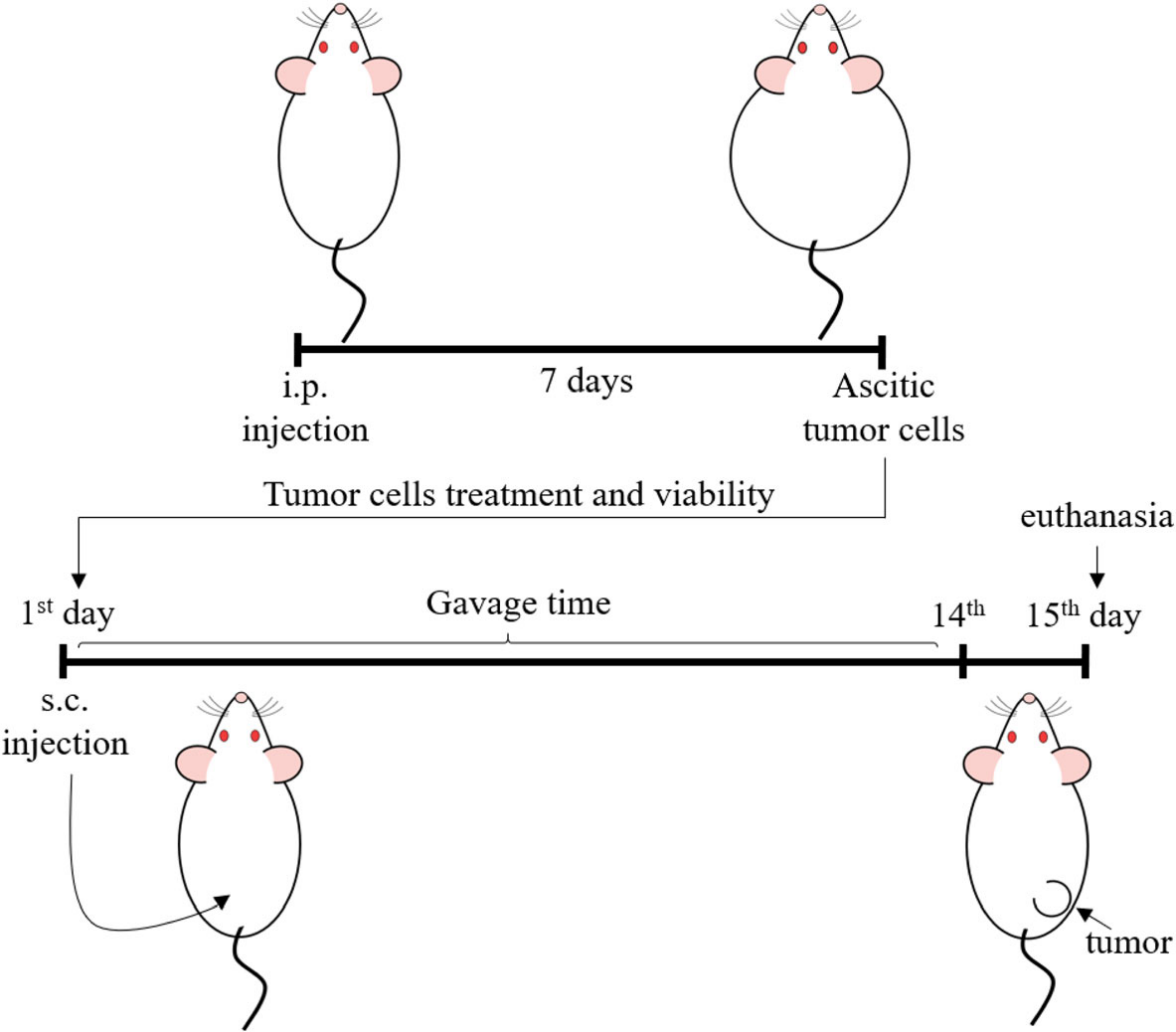
Experimental model of solid Ehrlich tumor and mice treatment with Fr-BuVt. Conditions: see materials and methods. Figure drawn by Morgan, H.J.N

The mice were randomly divided into the experimental groups (*n* = 10/group). All animals and cages were properly assigned to avoid potential confounders. Control groups treated with saline (NaCl 0.9%, 1 ml/kg) (CS) or 200 mg/kg Fr-BuVt (CV200) and Ehrlich tumor groups treated with saline (NaCl 0.9%, 1 ml/kg) (TS) or 200 mg/kg of Fr-BuVt (TV200). Blood glucose level and body weight were measured before de inoculation of Ehrlich cells or saline. Tumor inoculations were always done in the morning (8-9 h). Fr-BuVt or saline were administered orally by gavage once a day, at the same time (13-14 h) during 14 consecutive days after the inoculation (from the 1st to the 14th day). On the 15th day after inoculation the animals were submitted to experiments and/or euthanasia for collection of material, always in the morning (8-9 h) (Fig. 1). Body weight and food intake was measured every day using a precision electronic balance (Shimadzu ATY224), during the treatment. Food intake was calculated by the amount of food, in grams, per 100 g of animal per day.

### Euthanasia and ethical committee approval

After 15th days of tumor inoculation and treatment, mice were placed in a hermetic chamber with a high CO_2_ flow until the loss of pupillary reflex. The unconscious mice were decapitated using a guillotine proper to small animals (Insight, São Paulo, Brazil). All the experiments reported in this study were approved by the Ethics Committee on Animal Experiments (CEUA), UNESP, Bauru (Process number: 526/2017; volume 1).

### Tumor assessment

Ehrlich tumor animals were randomly divided into 4 groups (*n* = 10/group), and treated with saline (NaCl 0.9%, 1 ml/kg) (TS) or Fr-BuVt at different doses, 100 (TV100), 200 (TV200) or 400 mg/kg (TV400). The tumor length and width were measured with a digital caliper (Digimess) every 2 days starting on the seventh day after tumor inoculation in all Ehrlich tumor groups. Tumor volume was calculated using the following equation: volume (mm^3^) = width^2^ × length × π/6 [22]. All measures were taken from the same examiner to minimize bias. Percentage of tumor inhibition was calculated by taking tumor volume of day of all the groups as 100% and then percentage inhibition was calculated by comparing 0th day tumor volume with 3rd, 6th, and 9th day tumor volumes of the respective groups. After mice euthanasia the tumors were carefully removed and weighed on a precision electronic balance (Shimadzu ATY224).

### Acute toxicity test

Male Swiss mice were randomly divided into 2 groups (*n* = 10/group) and fasted for 4 h. Control group (CTL) received saline (NaCl 0.9%, 1 ml/kg) or 2000 mg/Kg Fr-BuVt (EXT) by gavage. The Fr-BuVt concentration was 10 times higher than the most effective treatment (200 mg/kg). The acute toxicity test was performed as described by Camaforte et al. [23].

### Tissue collection and evaluation of each organ

After 14 days of treatment (15th day) the animals were euthanized according to ethical committee. Liver, spleen, kidney, epididymal white adipose tissue, heart, gastrocnemius, extensor digitorum longus (EDL), tibialis and soleus skeletal muscle have been carefully removed and weighted in a precision electronic balance (Shimadzu ATY224). The relative weight of each tissue/organ was calculated by follow formula: tissue weight (g)/animal body weight (kg). Body weight ratio (%) was calculated as the formula: [(weight at the 15th day (g) × 100)/weight at the 1st day (g)] - 100.

### Biochemical analysis

At the end of treatment period, fasted (8-10 h) animals were euthanized and blood samples were collected and centrifuged to obtain the serum. Total proteins, albumin, triglycerides, ALT, AST, total cholesterol and HDL cholesterol were measured by commercial kits (BioSystems, Spanish) in A15 automatic spectrophotometer (BioSystems Analyzer) according to manufacturer’s instructions. Lactate was quantified using an YSI 2900 analyser (YSI, Ohio, USA). IL-6 and TNF-α were measured by specific ELISA (BioTek-PowerWave XS) Kit (R&D Systems). Free fatty acids were measured by ELISA previously described by Itaya & Ui [24]. Fasting blood glucose was measured in the 1st, 7th and 15th day of treatment using a glucometer (One Touch, Johnson & Johnson). Hepatic and muscular glycogen content were measured as previously described by Rafacho et al. [25].

### Protein expression by Western blot

Tumors of TV200 group (200 mg/kg Fr-BuVt) were collected in extraction buffer and Western blot was performed as described Camaforte et al. [23]. Images were acquired by documental system (G:BOX, Syngene) and analyzed using ImageJ software. Protein expression was corrected by expression of the loading control protein β-actin. The phosphorylated forms were corrected for the total forms only when the total shapes did not vary. Santa Cruz (São Paulo, Brazil) primary antibodies used (1:1000): BAX (sc-493), BCL-2 (sc-492), Caspase3 (sc-7148), ERK1/2 (sc-292,838), mTOR (sc-8319), NFκB (sc-372), PCNA (sc-56), p-EKR1/2 (sc-7383), p-mTOR (sc-101,738), p-NFκB (sc-101,749) and β-actin (sc-130,656).

### Statistical analysis

Data are presented as means ± standard error of mean (SEM). The results were submitted to adequate statistical analysis by SigmaStat Software (SPSS, Chicago, IL, USA). *p* values were calculated using the Student’s t test for normally distributed data and ANOVA followed by *Holm-Sidak* rank sum test for nonnormally distributed data. Differences were considered significant when *p* < 0.05.

## Results

### Identification of constituents from n-butanol fraction

**Fig. 2:**
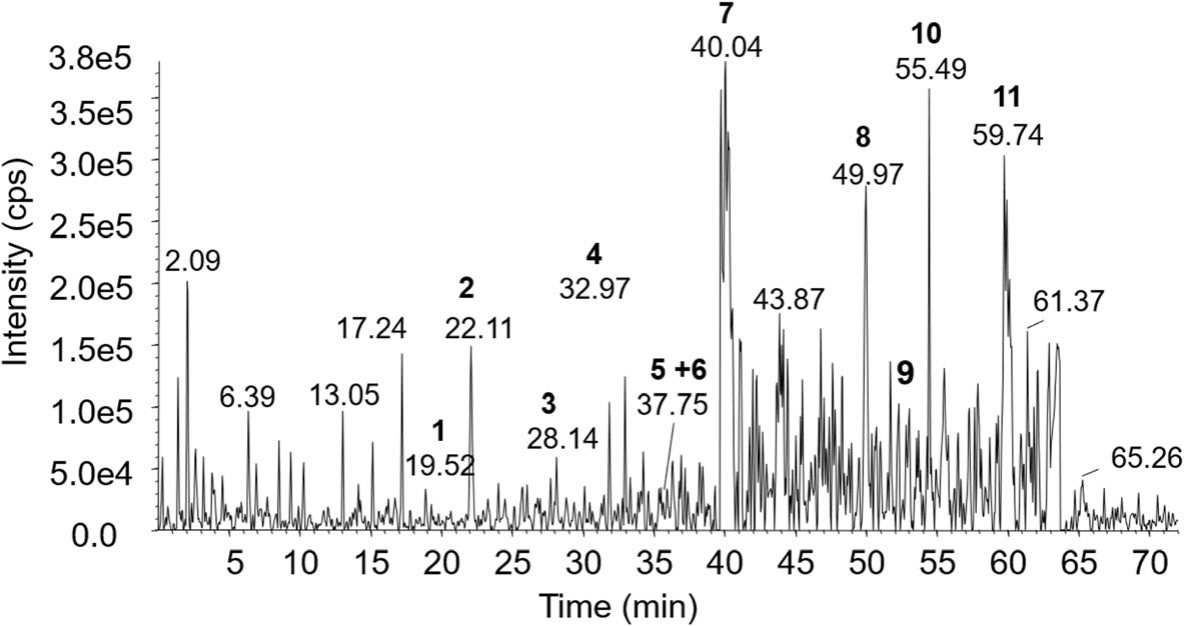
Base peak chromatogram obtained by HPLC-ESI-MS/MS from the Fr-BuVT of the leaves of *V. tucanorum* in negative mode. Conditions: see materials and methods

The constituents from the Fr-BuVt were identified based on experimental retention time and MS/MS data (Fig. 2, Table 1) obtained in comparison with available data in literature [9, 28]. Using this method, six compounds were identified: luteolin metyl ether *O*-hexoside (2), luteolin *O*-hexosideo (3) tetrahydroxy oleanoic *O*-hexoside (arjunglicoside) (7), pentahydroxy oleanoic acid (8), tetrahydroxy oleanoic acid (Sericic acid) (9), tetrahydroxy oleanoic acid derivative (10).

**Table 1:** Identification of constituents from the Fr-BuVT of *V. tucanorum* via LC-MS in negative mode

Peak	Retention time (min)	Molecular ion (***m/z***) [M-H]^**−**^	MS/MS ions (***m/z***)	Putative compound	Ref.
1	19.52	385	–	NI	–
2	22.11	461	299, 285, 283, 223	Luteolin metil ether *O*-hexoside	[9]
3	28.14	447	285	Luteolin *O*-hexoside	[9]
4	32.97	637	–	NI	–
5	36.47	727	–	NI	–
6	37.75	711	503	NI	–
7	40.04	665	503, 241	Tetrahydroxy oleanoic acid *O*-hexoside (Arjunglucoside I)	[8, 26]
8	49.97	519	501, 409, 301	Pentahydroxy oleanoic acid (Bellericagenin B)	[26]
9	54.44	503	485, 457, 409, 393	Tetrahydroxy oleanoic acid (Sericic acid)	[27]
10	55.49	571	503, 241	Tetrahydroxy oleanoic acid derivative	[27]
11	59.74	571	503	NI	–

*NI* Non-identified.

The fragmentation of the molecular ion at *m/z* 461 [M - H]^−^ (Peak 2) generated the fragment ions at *m/z* 299 [M - 162 - H]^−^ (loss of hexose), 285 e 283. The fragmentation of the molecular ion at *m/z* 447 [M - H]^−^ (Peak 3), presented the ion products at *m/z* 285 [M - 162 - H]^−^ (loss of hexose). This pattern of fragmentation was compatible with the flavones luteolin methyl ether *O*-hexoside and luteolin *O*-hexoside, respectivelly, previously identified in *V. tucanorum* leaves [9].

The fragmentation of the molecular ion at *m/z* 665 [M - H]^−^, (Peak 7) generated product ions at *m/z* 503 [M - 162 - H]^−^ (loss of hexose) and at *m/z* 241 (retro-Diels-Alder of C ring) and was identified as tetrahydroxy oleanoic *O*-hexoside (Arjunglucoside I), previously isolated from the leaves of *V. tucanorum* and *Qualea* spp. [8, 26]. The molecular ion at *m/z* 503 [M - H]^−^ (Peak 11) produced fragments ions at *m/z* 485, 457, 409 and 301 was identified as tetrahydroxy oleanoic acid (Sericic acid), previously isolated from *Vochysia pacifica* [27]. Fragmentation of the molecular ion *m/z* 571 [M - H]^−^ (Peak 12) with ion products at *m/z* 503 [M - 68 - H]^−^ and at *m/z* 241, confirms the presence of tetrahydroxy oleanoic acid derivative [27]. The molecular ion at *m/z* 519 [M - H]^−^ (Peak 9) presented the product ions at *m/z* 501, 409, 301 and was identified as pentahydroxy oleanoic acid (Bellericagenina B), previously identified in *Qualea* spp. (Vochysiaceae) [26].

### Evaluation of tumor growth and body weight

To determine the dose of Fr-BuVt used in the experiments, we performed a treatment with three different doses: 100 (TV100), 200 (TV200) and 400 mg/kg (TV400).

**Fig. 3:**
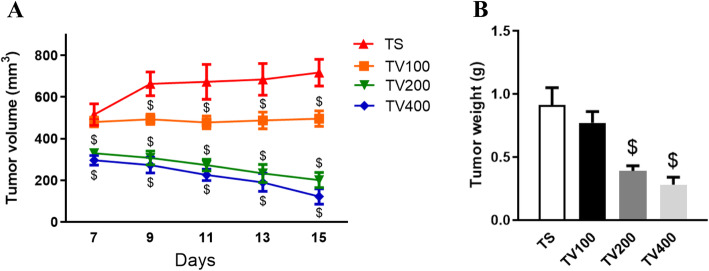
Tumor volume (**a**) and tumor weight (**b**) in Ehrlich tumor-bearing mice treated with Fr-BuVt (TV100, TV200 and TV400) or saline (TS). Data are means ± SEM, one-way ANOVA followed by Holm-Sidak post hoc test, *p* < 0.05 ($ vs TS)

The tumor volume was significantly lower in all doses from 9th day after inoculation compared to Ehrlich tumor mice treated with saline (TS), but just TV200 and TV400 groups showed expressive differences since the 7th day after inoculation (Fig. 3a). The lower tumor growth in mice treated with Fr-BuVt is also seen by the significant decrease of tumor weight in TV200 and TV400 groups at the end of treatment compared to Ehrlich tumor mice treated with saline (Fig. 3b).

Body weight at 15th day after inoculation showed no significant difference between groups. However, the TS group presented a reduction of 5.29% of the weight after 14 days of tumor inoculation compared with the first day. The TV200 and TV400 groups showed no weight reduction during treatment (Table 2).

**Table 2:** Body weight at the beginning (day 1), middle (day 7) and final (day 15) of the treatment with 100 mg/kg (TV100), 200 mg/kg (TV200) or 400 mg/kg (TV400) of Fr-BuVt

	Day 1 (g)	Day 7 (g)	Day 15 (g)	Weight ratio (%)
CS	45.35 ± 1.07	46.72 ± 1.11	47.82 ± 1.31	+ 5.44
TS	47.01 ± 1.17	47.55 ± 1.27	44.52 ± 1.08	− 5.29
TV100	46.79 ± 1.00	47.14 ± 1.23	46.07 ± 1.12	− 1.53
TV200	46.53 ± 1.23	48.15 ± 1.14	48.99 ± 1.17	+ 5.28
TV400	47.93 ± 1.4	49.13 ± 1.39	50.09 ± 1.49	+ 4.5

*CS* (Control animals treated with saline; *TS* (Ehrlich carcinoma-bearing mice treated with saline); TV100 (Ehrlich carcinoma-bearing mice treated with 100 mg/kg Fr-BuVt); TV200 (Ehrlich carcinoma-bearing mice treated with 200 mg/kg Fr-BuVt); TV400 (Ehrlich carcinoma-bearing mice treated with 400 mg/kg Fr-BuVt). Values are expressed as means ± SEM. There are no significative differences between groups in the same day, one-way ANOVA followed by *Holm-Sidak* post hoc test.

From the data analyzed, the dose of 200 mg/kg (TV200) was chosen to give continuity of the present study, since this group showed a significant improvement in the parameters analyzed so far. The treatment with 100 mg/kg (TV100) did not show significant improvements in body and tumor weights, while the concentration of 400 mg/kg (TV400) did not produced effects of greater intensity compared to the treatment with 200 mg/kg (TV200).

### Fr-BuVt did not show acute toxicity

The acute toxicity test was performed using a dose of 2000 mg/kg, ten times greater than that elected to follow up the study. Fr-BuVt administered to normal mice (EXT) did not provoke deaths or changes in the behavioral parameters analyzed (posture, secretions, and presence of convulsions) when compared with the group who received saline (CTL). There were no differences in body or organs weight of the animals in both groups (Table 3).

**Table 3:** Effect of the acute administration of saline (1 ml/kg) or Fr-BuVt (2000 mg/kg) on organs and body weight of male Swiss mice

	CTL	EXT
Spleen (g/kg)	4.39 ± 0.18	4.01 ± 0.15
Heart (g/kg)	4.82 ± 0.11	4.72 ± 0.14
Liver (g/kg)	51.55 ± 1.03	49.37 ± 1.06
Lung (g/kg)	7.8 ± 0.39	7.06 ± 0.37
Kidney (g/kg)	12.38 ± 0.31	12.77 ± 0.42
Initial body weight (g)	40.86 ± 1.12	41.37 ± 1.73
Final body weight (g)	44.2 ± 1.34	46.36 ± 1.65

*CTL* (Control; animals treated with saline); *EXT* (Extract; animals treated with Fr-BuVt). Values are expressed as means ± SEM. There are no significative differences, Student’s 푡t-test.

### Fr-BuVt regulated apoptosis and proliferation proteins

**Fig. 4:**
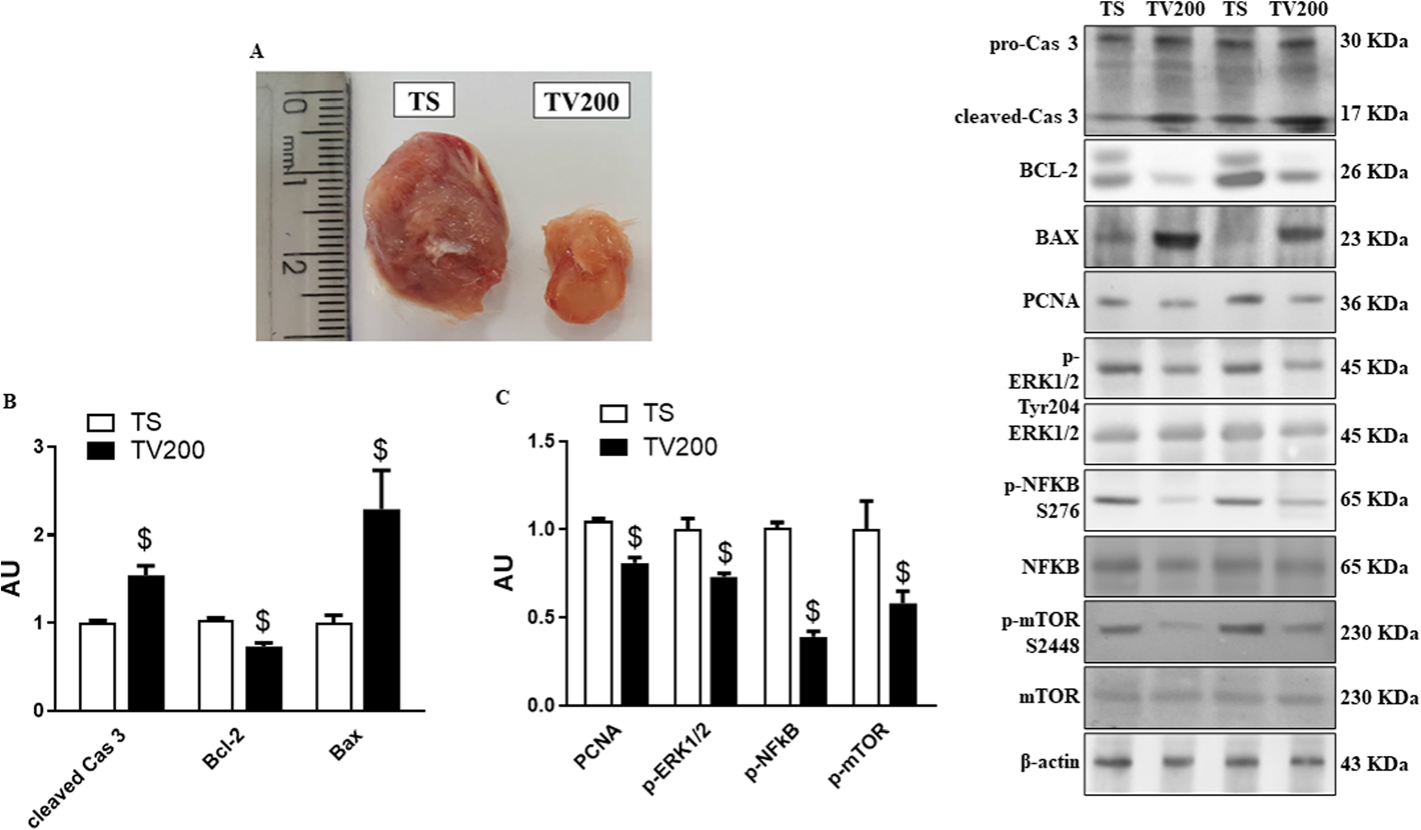
Tumor size (**a**) and protein content of cellular apoptosis (**b**) and proliferative (**c**) proteins by Western blot analysis in tumor tissue of animals treated with 200 mg/kg of Fr-BuVt (TV200) or saline (TS). β-actin was used as loading control and for phosphorylated proteins was used its total forms. Data are means ± SEM, Student’s 푡t-test, *p* < 0.05 ($ vs TS). Full-length blots are presented in Supplementary Fig. 1

To start the studies about how Fr-BuVt (TV200; 200 mg/kg) was able to reduce solid Ehrlich tumor size (Fig. 4a) we analyzed the contented and phosphorylation levels of key-proteins in tumor tissue after 14 days of treatment by Western blot. The data show an increase of the apoptotic, besides reduction of the proliferation-related proteins in the tumor cells in mice treated with Fr-BuVt (TV200) compared with TS animals. The apoptotic process stimulation is evidenced by the increase of the cleaved (active) form of caspase 3, as well as the reduction of Bcl-2 content and increase in BAX in TV200 group when compared with TS group (Fig. 4b). The antiproliferative activity of the fraction is evidenced by the significant reduction of PCNA, p-ERK1/2, p-NFκB and mTOR in TV200 compared with TS (Fig. 4c).

### Fr-BuVt prevents the installation of cachexia

**Fig. 5:**
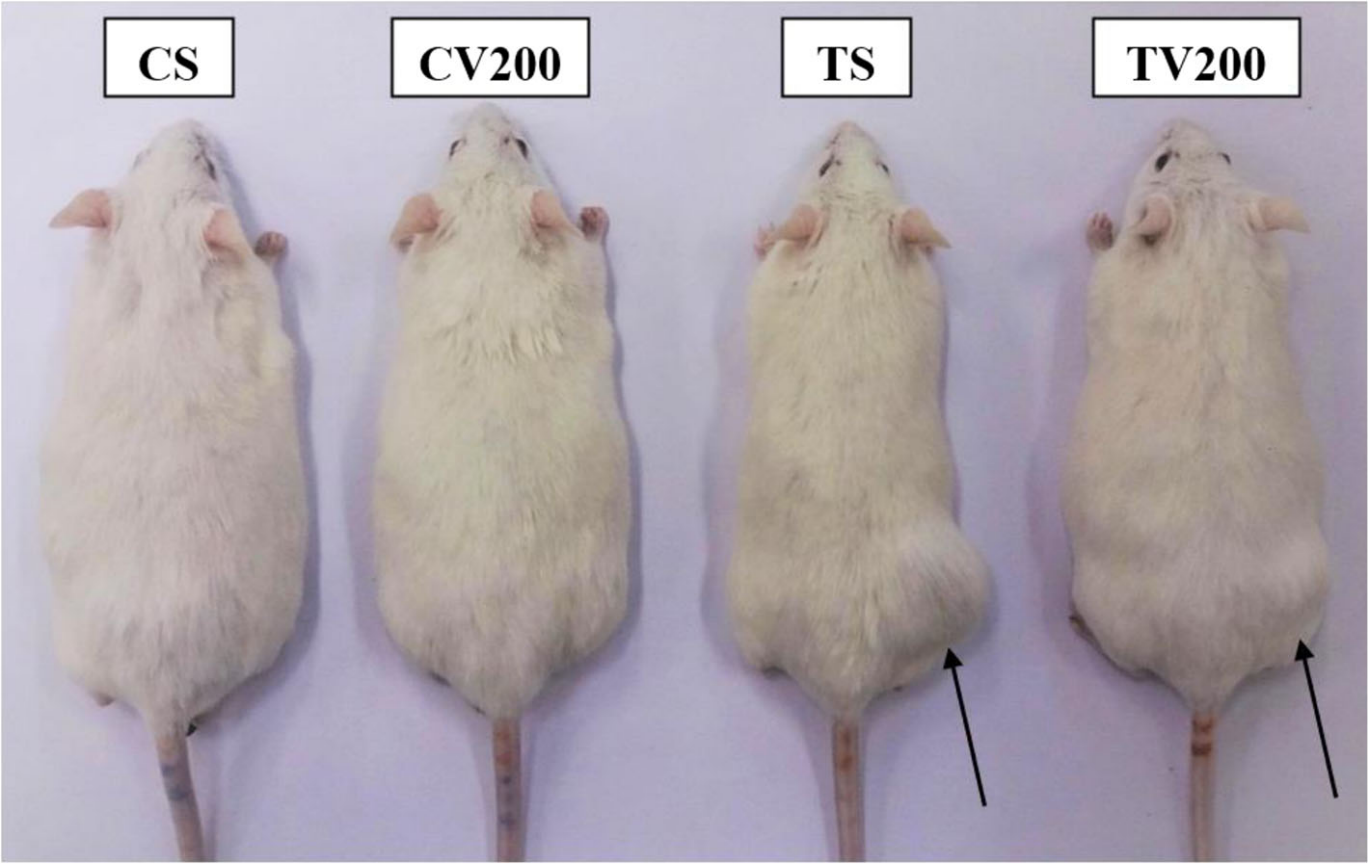
Representative figure of mice from control and tumor group treated with saline (CS and TS) or 200 mg/kg Fr-BuVt (CV200 and TV200). The arrow indicates the tumor

There were no significant differences in body weight and food intake between the experimental groups during the treatment period. However, on the 15th day of the treatment, the Ehrlich tumor animals treated with saline (TS) had a 5.46% decrease in weight compared to the 1st day, while tumor mice treated with Fr-BuVt (TV200) had a 6.41% increase (Table 4). In Fig. 5 it is possible to visualize that a representative animal of the TS group is leaner in relation to the other groups with a larger tumor as to the TV200 group.

**Table 4:** Body weight in the beginning (day 1), middle (day 7) and final (day 15) of the treatment and food intake of the control groups (CS and CV200) and Ehrlich tumor groups (TS and TV200)

	Day 1 (g)	Day 7 (g)	Day 15 (g)	Weight ratio (%)	Food intake (g/100 g)
CS	47.05 ± 1.29	49.86 ± 1.43	49.91 ± 1.34	+ 6.07	18.11 ± 0.37
CV200	46.79 ± 0.91	48.27 ± 0.91	48.95 ± 0.61	+ 4.61	20.22 ± 1.25
TS	47.56 ± 1.24	48.98 ± 1.15	44.96 ± 1.14	−5.46	19.4 ± 0.8
TV200	46.92 ± 1.51	48.25 ± 1.65	49.93 ± 1.82	+ 6.41	18.49 ± 0.51

*CS* (Control animals treated with saline); *CV200* (Control animals treated with 200 mg/kg Fr-BuVt); TS (Ehrlich carcinoma-bearing mice treated with saline); TV200 (Ehrlich carcinoma-bearing mice treated with 200 mg/kg Fr-BuVt). Values are expressed as means ± SEM. There are no significative differences, ANOVA followed by *Holm-Sidak* post hoc test.

**Fig. 6:**
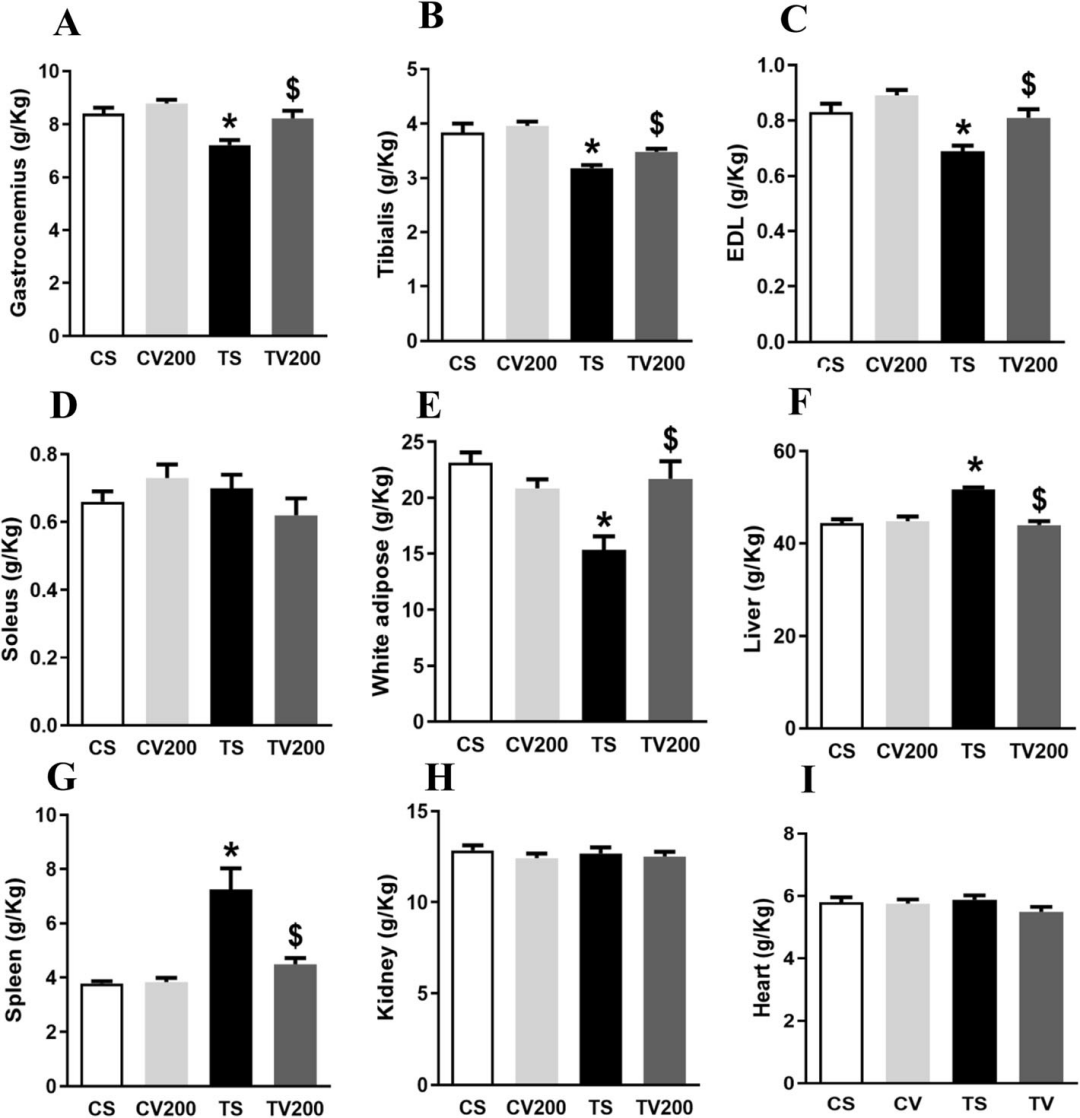
Gastrocnemius (A), tibialis (B), EDL (C), soleus (D), white adipose tissue (E), liver (F), spleen (G), kidney (H) and heart (I) weights after 14 days of treatment with saline or 200 mg/kg Fr-BuVt in control (CS e CV200) or tumor groups (TS e TV200). The weights were corrected for the body weight of each animal. Data are means ± SEM, two-way ANOVA followed by Holm-Sidak post hoc test, *p* < 0.05 (* vs CS; $ vs TS)

While Ehrlich tumor animals treated with Fr-BuVt (TV200) had no difference in organ weight compared to control animals, tumor mice treated with saline (TS) had the weights of glycolytic muscles gastrocnemius (Fig. 6a), tibialis (Fig. 6b) and EDL (Fig. 6c) decreased. However, in the oxidative soleus muscle (Fig. 6d) difference between the experimental groups was not observed. In addition, TS group showed a decrease in white adipose tissue weight (Fig. 6e) compared to the control group (CS). Liver (Fig. 6f) and spleen weights (Fig. 6g) of TS animals showed a significant increase in relation to CS group. Fr-BuVt treatment in Ehrlich mice prevented these alterations in organs weights that were observed in tumor-bearing animals treated with saline (TS), since there was no difference when comparing TV200 group with control group (CV200). Kidneys and heart weights (Fig. 6h; Fig. 6i) did not show significant difference between the experimental groups.

### Fr-BuVt improved metabolic parameters of cachectic mice

Ehrlich carcinoma-bearing mice showed increased serum levels of free fatty acids (FFA), triglycerides (TG), ALT, AST and lactate, while total proteins, albumin, HDL and total cholesterol (Table 5) show significant decrease compared to control group (CS). The treatment with 200 mg/kg Fr-BuVt (TV200) prevented all these alterations in metabolic parameters observed in TS group. There was no difference between TV200 and CV200 groups in any of these analyses.

**Table 5:** Metabolic parameters of the control and tumor groups treated with saline (CS and TS) or 200 mg/kg Fr-BuVt (CV200 and TV200)

	CS	CV200	TS	TV200
Initial glycaemia (mg/dL)	80.3 ± 1.7	79.0 ± 3.2	82.4 ± 1.4	81.1 ± 2.0
Final glycaemia (mg/dL)	80.0 ± 4.4	77.5 ± 4.4	59.4 ± 2.9 *	81.7 ± 4.0 ^$^
Triglycerides (mg/dL)	107.0 ± 5.0	97.6 ± 4.3	189.7 ± 10.6 *	106.2 ± 5.9 ^$^
FFA (mEq/L)	0.55 ± 0.06	0.42 ± 0.02 *	0.81 ± 0.04 *	0.56 ± 0.05 ^$^
Total cholesterol (mg/dL)	121.5 ± 4.6	120.1 ± 5.9	101.3 ± 5.5 *	118.7 ± 3.2 ^$^
HDL cholesterol (mg/dL)	74.6 ± 2.8	82.2 ± 2.9	52.9 ± 1.3 *	70.6 ± 3.8 ^$ &^
ALT (U/L)	79.1 ± 3.8	73.2 ± 6.7	144.1 ± 8.7 *	81.0 ± 4.06 ^$^
AST (U/L)	286.1 ± 20.2	299.2 ± 24.0	439.0 ± 22.2 *	281.1 ± 16.1 ^$^
Total proteins (g/dL)	6.1 ± 0.1	5.8 ± 0.3	5.1 ± 0.1 *	5.9 ± 0.1 ^$^
Albumin (g/dL)	1.8 ± 0.05	1.92 ± 0.08	1.4 ± 0.05 *	1.7 ± 0.05 ^$^
Lactate (mmol/L)	9.5 ± 0.5	9.28 ± 0.65	12.5 ± 0.9 *	9.4 ± 0.4 ^$^
Hepatic glycogen (g/100 g)	2.9 ± 0.3	2.5 ± 0.4	0.34 ± 0.04 *	2.5 ± 0.2 ^$^
Muscle glycogen (g/100 g)	0.81 ± 0.06	0.85 ± 0.09	0.44 ± 0.07 *	0.68 ± 0.06 ^$^
TNF-α	1.18 ± 0.2	1.0 ± 0.1	9.7 ± 0.9 *	1.6 ± 0.2 ^$^
IL-6	3.4 ± 0.4	3.0 ± 0.5	22.5 ± 0.7*	4.0 ± 0.4 ^$^

Values are expressed as means ± SEM, two-way ANOVA followed by *Holm-Sidak* post hoc test, *p* < 0.05 (* vs CS; $ vs TS; & vs CV).

Initial fasting blood glucose (1st day) was not different among the experimental groups. Final fasting blood glucose (15th day) is decreased in TS group compared with CS and TV200 groups, and TS group had a significant reduction compared to its initial value (1st day). Ehrlich carcinoma-bearing mice animals treated with Fr-BuVt (TV200) did not show difference compared with control animals (CV200) (Table 5).

The levels of both liver and muscle glycogen in TV200 group were not different when compared with control group (CV200), but are significantly higher compared to TS group. The TS group showed significant decrease in glycogen contents both in liver and muscle compared to CS group (Table 5).

The serum cytokines TNF-α and IL-6 were significantly increased in TS group compared to CS and TV200 groups. The TV200 group did not show significant difference compared to CV200 (Table 5).

## Discussion

The use of plants for the treatment of diseases is a millenary culture of humanity. The secondary metabolites present a number of interesting biological activities to combat many diseases [29]. In this study, we evaluated the potential of the butanolic fraction of leaves of *Vochysia tucanorum* Mart. (Fr-BuVt) in cancer cachexia treatment, using Ehrlich carcinoma-bearing mice as a model. We identify in Fr-BuVt mainly triterpenes such as oleanolic acid, and flavones such as luteolin.

Even though natural products may represent effective alternatives in the treatment of various diseases, it can also be a problem for health [30]. Ethnobotanical studies report the risks of intoxication and the side effects of those compounds [31, 32]. Gomes et al. (2009) demonstrated the absence of acute toxicity with single oral administration of 5000 mg/kg of *V. tucanorum* methanolic leaf extract. However, the acute toxicity of Fr-BuVt had not been analyzed. A single oral administration of 2000 mg/kg of Fr-BuVt did not produce any sign of acute toxicity in animals. These data allowed the continuation of further pharmacological studies with this medicinal plant.

The treatment with Fr-BuVt (200 mg/kg) during 14 days did not produced alteration in serum AST and ALT levels, and kidney weight, which indicates that Fr-BuVt (200 mg/kg) does not appear to present liver and kidney toxicity, which are common in many studies with natural products [31, 33].

Those triterpenes identified in Fr-BuVt have been described in literature as anti-inflammatory and anticancer compounds [9–11]. The observed decrease in tumor volume and weight indicates that Fr-BuVt have antitumoral activity in vivo in Ehrlich carcinoma-bearing mice. All the three doses tested of Fr-BuVt were able to reduce tumor growth, but only the doses of 200 and 400 mg/kg were able to reduce tumor weight, and prevent loss of body weight. The treatment with 100 mg/kg of Fr-BuVt (TV100) did not show improvement in body weight and tumor weight. Therefore, the dose of 200 mg/kg Fr-BuVt was used in our next analyses.

Then, we investigated proteins involved in apoptosis and cell proliferation pathways in order to identify possible mechanisms of Fr-BuVt action in the Ehrlich tumor. The literature reports that pentacyclic triterpenes, like betulinic acid can induce apoptosis in neoplastic cells by activating the caspase pathway through the release of cytochrome c, and therefore it can regulate antiapoptotic proteins like Bcl-2 and proapoptotic proteins like BAX [34]. Our data show that Ehrlich mice treated with Fr-BuVt have significant increase in cleaved caspase 3 expression in tumor tissue compared with tumor from animals treated with saline. In addition, expression of Bcl-2 was reduced and that of BAX increased, corroborating the mechanisms of action of these compounds described in the literature.

Caspases, a family of cysteine proteases important in apoptosis program, are synthesized as inactive proenzymes and become activated upon cleaved. Caspase 3 is a member of effector apoptosis subfamilies, the “executioner” class [35, 36]. In this aspect, while Bcl-2 inhibit apoptosis, BAX induces this process through regulation of mitochondrial membrane permeabilization. Mitochondrion-targeted agents represent a great and promising therapy as a novel class of anticancer drugs [37]. Thus, our data suggest the regulation of apoptotic process is probably one of Fr-BuVt mechanisms against tumor cells.

Besides the apoptotic process control, an anticancer therapy which regulates proliferation pathways may be interesting. Studies have been reported that triterpenes are able to regulate them [38, 39]. We investigate the expression and phosphorylation of some proteins involved in cellular proliferation. Our data show that Fr-BuVt can suppress mTOR, ERK, NFκB and PCNA expressions in Ehrlich carcinoma-bearing mice tumor.

PCNA is a marker of cell proliferation, coordinating the DNA replication machinery, and a range of cell functions related to survival and maintenance such as cell growth and death [40]. Lai et al. [41] demonstrated that Brucein D, a tetracyclic triterpene, has anti-proliferative activity in pancreatic cancer cell line through down-regulation of PCNA and Ki-67, important markers of cell proliferative activity.

Checker et al. [42] demonstrated that ursolic acid, a pentacyclic triterpenoid, has a potent anti-inflammatory activity. This molecule can suppress NFκB activation an inhibit phosphorylation of ERK in lymphocytes. NFκB family are transcription factors involved in regulation of many biological responses, and play an import role in regulate expression of genes involved in oncogenic process, such as proliferation, migration and apoptosis [43]. ERK pathway is determinant to several cell process such as proliferation, survival and differentiation. This pathway is up-regulated in many human tumors, being a target for anticancer drugs. The blockage of ERK can result in anti-proliferative, anti-metastatic and anti-angiogenic effects [44].

In addition, mTOR plays an important role in cancer therapies because it participates in cell growth, proliferation, autophagy and survival. mTOR inhibitors have been tested against tumor cells with promising results [45, 46]. Our results suggest that Fr-BuVt exhibits anticancer activity by increasing apoptosis through caspase and Bcl-2 pathways, and decreasing proliferation through NFκB, mTOR and ERK inhibition.

Although studies demonstrate anticancer activity of many molecules and extracts from plants, they do not evaluate the potential activity of those compounds in cachexia development. The difficult treatment of cachexia lead us to look for new approaches therapy that can treat cachexia and still have antitumor activities [4]. The medicinal plants become an interesting tool in this field and the Fr-BuVt presents a promising effect against tumor cells. The synergism between the triterpene molecules present in Fr-BuVt would be interesting for the treatment of cancer cachexia.

Violato et al. [21] showed that Ehrlich carcinoma-bearing mice exhibit cachectic state 14 days after tumor inoculation with significative loss of body weight, a common aspect also found in the present study. However, Frajacomo et al. [47] did not see alteration in body weight in this model, but they have found evidences that solid Ehrlich carcinoma reproduces functional and biological characteristics of the cachectic syndrome, which gives us assurance about the model used. Here we show that Ehrlich carcinoma-bearing mice present a catabolic metabolism as seen in cachexia and the treatment with Fr-BuVt can prevent this process.

The main characteristic of this syndrome is weight loss and muscle atrophy. It is interesting to note that, although there was no significant difference in body weight among the experimental groups, the tumor animals treated with saline (TS) showed a loss of more than 5% of body weight, while treatment with Fr-BuVt prevented the decrease of body weight. Cancer patients with weight loss greater than 5% are diagnosed with cachexia [2). Accompanied by the weight loss, Ehrlich tumor animals presented reduction of EDL, tibialis and gastrocnemius muscles weights, while soleus muscle keeps its weight. In cachexia there are selective loss of specific types of muscle fibers. Despite some doubts, apparently there is a preferential loss of type II fibers present in EDL, tibialis and gastrocnemius, with preservation of type I fibers observed in soleus [48]. However, the treatment with Fr-BuVt (TV200) prevents the loss of lean mass. Triterpenes have been describing as factors which are able to modulate skeletal muscle mass by improve protein synthesis and decrease proteolysis [13, 14].

Anorexia is often found in cancer patients and may occur in cachexia. Different from cachexia, anorexia leads to a greater loss of adipose tissue than muscle, and is associated with loss of appetite [3, 49]. When anorexia is present, it significantly worsens the cachexia. The fat atrophy occurs by the tissue response from tumor factors like LMF/ZAG and cytokines, which increase the lipolysis [3]. In our results, we have not seen difference in food intake among the experimental groups, despite the decrease of fat mass and increase of TG and FFA in Ehrlich tumor treated with saline (TS) suggest the increase of lipolysis process in this model. In contrast, the treatment with Fr-BuVt prevented the reduction of white adipose tissue weight as well as TG and FFA increase.

The lean and fat weight loss along with other metabolic parameters such as liver and muscle glycogen, decrease of glycaemia and serum proteins, as well as increase of serum lactate are characteristics of a catabolic process that should be occurring in the TS group. As our data shows, the treatment with Fr-BuVt had a very potent action in preventing this catabolic state installation in Ehrlich carcinoma-bearing mice.

Violato et al. [21] demonstrated that Ehrlich tumor presence leads to an imbalance in insulin secretion and reduction of insulinaemia and glycemia besides increase of glucose tolerance and insulin sensitivity. This imbalance in insulin metabolism in Ehrlich carcinoma-bearing mice can worsen the cachectic state, and be one of the explanations for the atrophy of lean and fat mass, since insulin is an anabolic hormone important for the maintenance of muscle mass and adipose tissue. Our results corroborate with those data and TS group present hypoglycemia after 15 days. The treatment with Fr-BuVt (TV) could prevent the hypoglycemia.

The role of inflammation and cytokines has been discussed as being one of the main factors for the establishment of cachexia, in especial involving TNF-α and IL-6 [3, 6]. In general, these pro-inflammatory cytokines have catabolic effect involving the energetic metabolism. For example, in skeletal muscle, TNF-α can induce proteolysis by ubiquitin-proteasome system, besides the increase in hepatic gluconeogenesis and loss of adipose tissue [50]. Our data shows that Ehrlich carcinoma-bearing mice had increased IL-6 levels, as well as gain of spleen mass, suggesting the presence of inflammation. On the other hand, the treatment with Fr-BuVt improved those parameters, indicating a possible anti-inflammatory activity of the fraction.

Ehrlich carcinoma-bearing mice (TS) had increase of serum ALT and AST as well as increase of liver weight, and plasma proteins and lipoproteins, suggesting that the tumor syndrome affected the liver functions alterations. According to the literature, liver dysfunction worsen the cachectic condition [2, 5]. Treatment with Fr-BuVt improves these parameters, thus acting on very decisive factors implicated in cachexia installation.

## Conclusions

In summary, our study demonstrates that the administration of Fr-BuVt to Erhlich carcinoma bearing mice exerts an important antitumor activity besides prevent the establishment of cachexia, ameliorating the parameters associated with this syndrome. This capacity of combating tumor cells is perhaps the most important activity of this fraction, since the reduction of tumor progression may be the factor responsible for preventing the establishment of the cachectic syndrome.

## Supplementary Information

**Additional file 1.**

## Data Availability

The datasets generated and/or analyzed during the study are available from the corresponding author on reasonable request.

## Abbreviations

Fr-BuVt: Butanolic fraction of *Vochysia tucanorum* Mart
NFκB: Factor nuclear kappa B
mTOR: Mammalian target of rapamycin
ERK: Extracellular signal–regulated kinasesin i
BAX: Bcl2-associated X protein
BCL-2: B-cell lymphoma 2
IL-6: Interleucina6
TNF-α: Tumor necrosis factor alpha
AIDS: Acquired immunodeficiency syndrome
UNBA: Herbarium of the department of biological sciences, school of sciences, bauru campus, São Paulo State university
HPLC: High performance liquid chromatography.
DP: Declustering potential
EP: Entrance potential
CEP: Cell entrance potential
EPI: Enhanced product ion
CEUA: Ethics committee on animal experiments
CS: Mice control group treated with saline
CV: Mice control group treated with Fr-BuVt (200 mg/kg)
TS: Ehrlich carcinoma-bearing mice group treated with saline
TV200: Ehrlich carcinoma-bearing mice group treated with Fr-BuVt (200 mg/kg)
TV400: Ehrlich carcinoma-bearing mice group treated with Fr-BuVt (200 mg/kg)
CTL: Mice control group treated with saline for acute toxicity test
EXT: Mice group treated with Fr-BuVt (2000 mg/kg) for acute toxicity test
ALT: Alanine transaminase
AST: Aspartate transaminase
HDL: High-density lipoprotein cholesterol
SEM: Standard error of mean
MS: Mass spectrometry
EDL: Extensor digitorum longus muscle
PCNA: Proliferating cell nuclear antigen
FFA: Free fatty acids
