# Supplementary material for: Vochysia tucanorum Mart. butanol fraction presents antitumoral activity in vivo and prevents the installation of cachexia in solid Ehrlich tumor model
Source: BMC Complement Med Ther. 2021 Jan 7;21:20. doi: 10.1186/s12906-020-03190-1 (PMC7791751; doi:10.1186/s12906-020-03190-1)
Supplement: Supplementary file 1 — Additional file 1. [file 12906_2020_3190_MOESM1_ESM.pptx]

## Slide 1
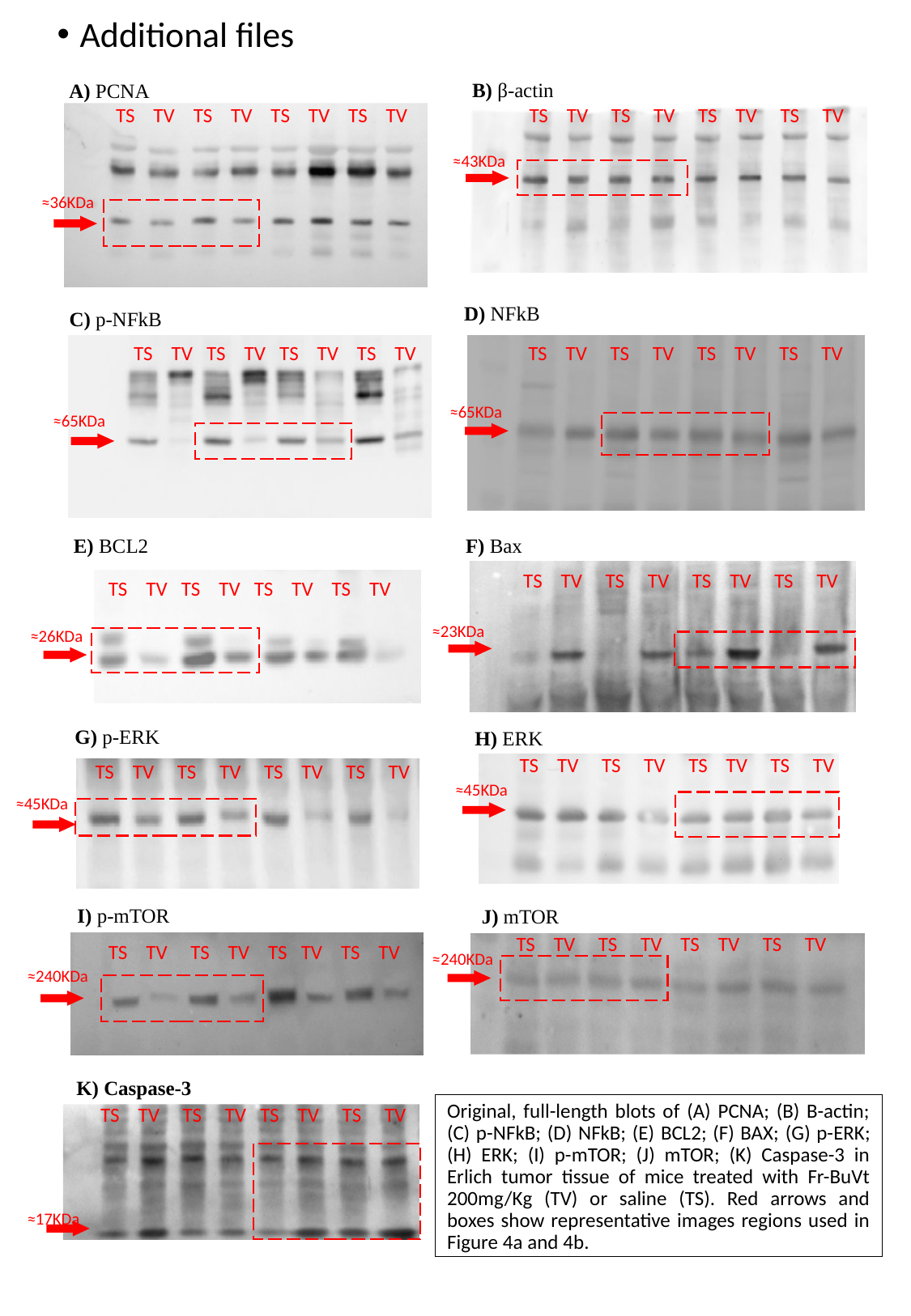

Additional files
B) β-actin
A) PCNA
TS TV TS TV TS TV TS TV
TS TV TS TV TS TV TS TV
≈43KDa
≈36KDa
D) NFkB
C) p-NFkB
TS TV TS TV TS TV TS TV
TS TV TS TV TS TV TS TV
≈65KDa
≈65KDa
E) BCL2
F) Bax
TS TV TS TV TS TV TS TV
TS TV TS TV TS TV TS TV
≈23KDa
≈26KDa
G) p-ERK
H) ERK
TS TV TS TV TS TV TS TV
TS TV TS TV TS TV TS TV
≈45KDa
≈45KDa
I) p-mTOR
J) mTOR
TS TV TS TV TS TV TS TV
TS TV TS TV TS TV TS TV
≈240KDa
≈240KDa
K) Caspase-3
Original, full-length blots of (A) PCNA; (B) B-actin; (C) p-NFkB; (D) NFkB; (E) BCL2; (F) BAX; (G) p-ERK; (H) ERK; (I) p-mTOR; (J) mTOR; (K) Caspase-3 in Erlich tumor tissue of mice treated with Fr-BuVt 200mg/Kg (TV) or saline (TS). Red arrows and boxes show representative images regions used in Figure 4a and 4b.
TS TV TS TV TS TV TS TV
≈17KDa
